# Application of clinical proteomics in acute respiratory distress syndrome

**DOI:** 10.1186/s40169-014-0034-1

**Published:** 2014-10-15

**Authors:** Maneesh Bhargava, LeeAnn Higgins, Christine H Wendt, David H Ingbar

**Affiliations:** Division of Pulmonary, Allergy, Critical Care and Sleep Medicine, University of Minnesota, Minneapolis, USA; Biochemistry, Molecular Biology and Biophysics, University of Minnesota, Minneapolis, USA; Minneapolis Veterans Affairs Medical Center, Minneapolis, MN USA

**Keywords:** Proteomics, Bioinformatics, ARDS, ALI, Biomarkers

## Abstract

Acute Respiratory Distress Syndrome (ARDS) is a devastating cause of hypoxic respiratory failure, which continues to have high mortality. It is expected that a comprehensive systems- level approach will identify global and complex changes that contribute to the development of ARDS and subsequent repair of the damaged lung. In the last decade, powerful genome-wide analytical and informatics tools have been developed, that have provided valuable insights into the mechanisms of complex diseases such as ARDS. These tools include the rapid and precise measure of gene expression at the proteomic level. This article reviews the contemporary proteomics platforms that are available for comprehensive studies in ARDS. The challenges of various biofluids that could be investigated and some of the studies performed are also discussed.

**Electronic supplementary material:**

The online version of this article (doi:10.1186/s40169-014-0034-1) contains supplementary material, which is available to authorized users.

## Introduction

Biological systems function via intricate orchestrated cellular processes in which various cellular entities participate in a tightly regulated manner. Proteins are the `work horse’ of the cell and alterations of their behavior often are implicated in the development of diseases. Due to limitations in technology most of the initial biomedical research to determine the structure-function of the proteins was performed one molecule at a time. Since the completion of the human genome project there has been increasing interest to study the broader changes of proteins within a biological system, a field defined as *Proteomics*[1]. Prior reviews have focused on current techniques available at that time as applied to interstitial lung diseases [2],[3], lung cancer [4]-[6] and other lung diseases [7]-[9]. Some of these reviews have described the principles of electrophoresis, the gel based methodologies and basic principles of mass spectrometry (MS) [7]. With improvements in the MS platforms, the proteomics research has grown substantially from simply identifying proteins present in a clinical sample to the capability for absolute and relative quantification of proteins by either LC-MS/MS or targeted proteomics. With these advances the field is now poised to identify candidate biomarkers and give insight into the biological mechanisms of disease. In this review, we highlight the principles and advances in proteomic platforms focusing on contemporary MS methodologies; discuss sample preparation challenges related to biofluids for pulmonary research and the application of current proteomic techniques in Acute Respiratory Distress Syndrome (ARDS).

### Proteomics methodologies

Traditional proteome analysis began with 2-dimensional (2D) SDS-PAGE protein separation and differential analysis of gel spot patterns [10],[11]. The advent of specialized methods for mass spectrometric detection of proteins and peptides that were made possible with the revolutionary ionization techniques matrix-assisted laser desorption ionization (MALDI) [12] and electrospray ionization (ESI) [13] advanced all proteome pursuits starting in the mid 1990’s [14]-[16].

#### Sample-specific details

Procurement of body fluid samples destined for proteomics projects must be controlled for protein loss, degradation, proteolysis and oxidative modifications [17]-[19]. Variability in sample handling should be minimized for quantitative analyses of protein expression levels to ensure conclusions are made based on biological variability not variability in sample handling. Wide dynamic ranges in protein abundances may limit or preclude detection limits for clinically interesting, low abundant proteins such as tissue leakage proteins and transcription factors [20],[21]. When protein dynamic range is wide (e.g., serum where protein abundance spans 10 orders of magnitude), high abundant protein depletion with spin cartridges or columns is often necessary to maximize protein detection [20]. Assessment of the reproducibility of depletion products, when employed, is critical for both qualitative and quantitative projects [22].

#### Top-down analyses

`Top-down’ analyses of proteins by MS employ measurements on intact proteins [23],[24]. Two common technologies, MALDI and surface enhanced laser desorption SELDI- time of flight (TOF), provide protein profiles but do not provide protein identification. Thus these have been utilized as screening methods for comparison of protein profiles from various sample types among populations of healthy and diseased patients for the pursuit of disease biomarker detection. Solid phase extraction (SPE) and chip-based techniques used for these top-down analyses are fast and efficient methods for intact protein purification, with the principal limitation that relatively small subsets of proteins are extracted and subsequently detected. SPE is employed for protein purification, desalting and concentration prior to MALDI-TOF MS detection. MALDI-TOF MS has been performed in both serum [25],[26] and bronchoalveolar lavage fluid [27] for biomarker discovery. In a variation to MALDI-TOF MS, surface enhanced selective protein capture, an affinity-based chip method for protein extraction prior to SELDI-TOF detection [28] has been used for biomarker discovery for subjects with pulmonary sarcoidosis.

#### Bottom-up analyses

**Figure 1:**
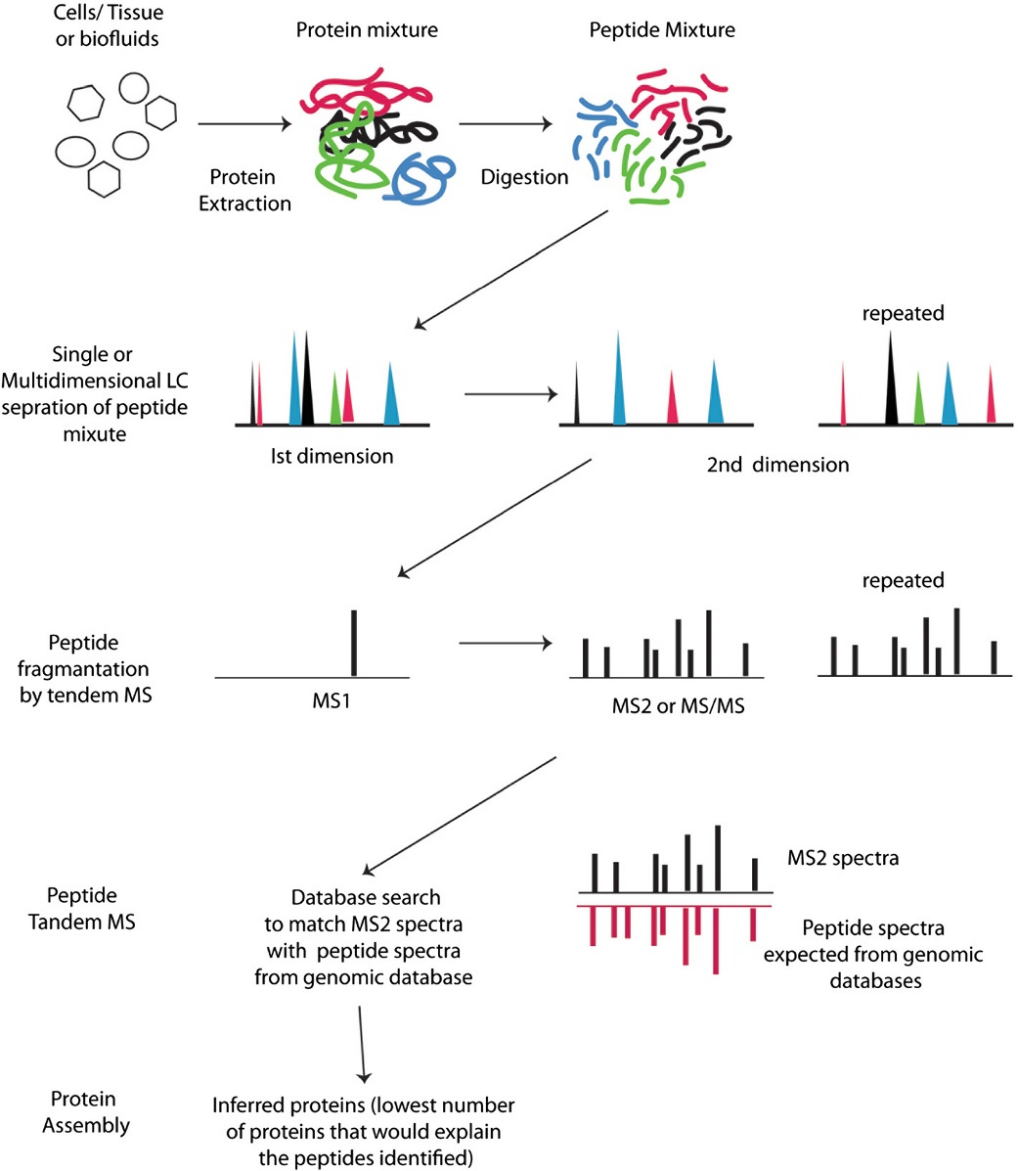
**Workflow of `bottom-up’ or shotgun proteomics.** Protein extracts from cells, tissue or biofluids are prepared by mechanical (e.g., glass bead or homogenization) or chemical-based (precipitation, detergent solubilization) methods. Proteins are proteolytically digested into peptides, usually with trypsin, that are separated by 1D or 2D chromatographic separation. The final chromatographic step is performed in-line with the mass spectrometer. Two scan types are acquired: MS1 spectra contain intact peptide mass to charge (m/z) values; MS2 or tandem MS (MS/MS) spectra represent peptide fragment ion m/z values. Peptide MS1 and MS2 data are correlated with theoretical peptide m/z values with database search programs that use protein sequences as templates; parsimonious protein identifications with peptide matches are reported.

In contrast to studying intact proteins***,*** analysis of peptide mixtures obtained after proteolytic treatment of protein mixtures is called `bottom-up’ or `shot-gun’ proteomics [29],[30]. `Bottom-up’ proteomics studies are typically implemented for discovery-based experiments that provide protein identification and can also provide relative and absolute protein quantitative measurements with the appropriate experimental design. Two basic workflows for bottom-up proteomic studies are: 1) solution-based proteolytic digestion of protein extracts [31]-[34] such as done for studies in ARDS by others [34],[35] and our laboratory [36]; 2) GeLC analysis, which entails one dimensional (1D) SDS-PAGE separation of proteins, excision of consecutive gel regions and proteolytic digestion of proteins in each gel section [14],[37],[38]. The steps in a `Bottom-up’ proteomic workflow are shown in Figure 1 and include 1) Proteolytic digestion 2) Chromatographic peptide separation 3) Peptide tandem MS 4) Database search for peptide identification and 5) Protein assembly.

#### Separation methods

Prior to MS protein identification and measurement peptide mixtures such as a protein from excised gel band could be separated by I-D liquid chromatography (LC) [39]. 2D- LC is used for fractionation of complex peptide mixtures such as tissue or cellular proteins [29]. The first dimension typically separates peptides based on peptide pI or hydrophobicity in high pH solvent. The second dimension separation is usually based on peptide hydrophobicity in low pH solvent and is performed `in-line’ with the MS-ESI interface between the column tip and MS orifice [40],[41]. In a less common approach, the second dimension LC eluent is directed onto a metal plate or target for LC MALDI-TOF analysis [42],[43].

#### Peptide and protein identification

Peptide mass spectra generated by tandem MS are used for protein identification in bottom-up experiments. Program-specific algorithms compare theoretically derived peptide fragment pattern (generated *in silico)* to experimental peptide data [44]-[46]. Potential peptide database matches are ranked, scored and reported. Highest scoring peptides are used to generate a list of inferred proteins present in the complex mixture (protein assembly). Parsimonious protein assembly is used so the lowest number of inferred proteins would account for the detected peptides [47],[48]. Variations on database search algorithms provide a multitude of commercial and open source search programs for database searching, each of which has a unique peptide candidate scoring scheme and protein inference method. One or more peptide matches per protein is sufficient evidence for detection of the protein in the sample [49]. False discovery rates (FDR) of protein identification are available when the target protein database is reversed or scrambled and concatenated to the target database [50]-[52]. Public, species-specific protein data repositories that contain translated genomic sequences provide templates for the software programs (e.g., http://www.ncbi.nlm.nih.gov/protein and http://www.uniprot.org/).

#### Quantitative proteomics

**Figure 2:**
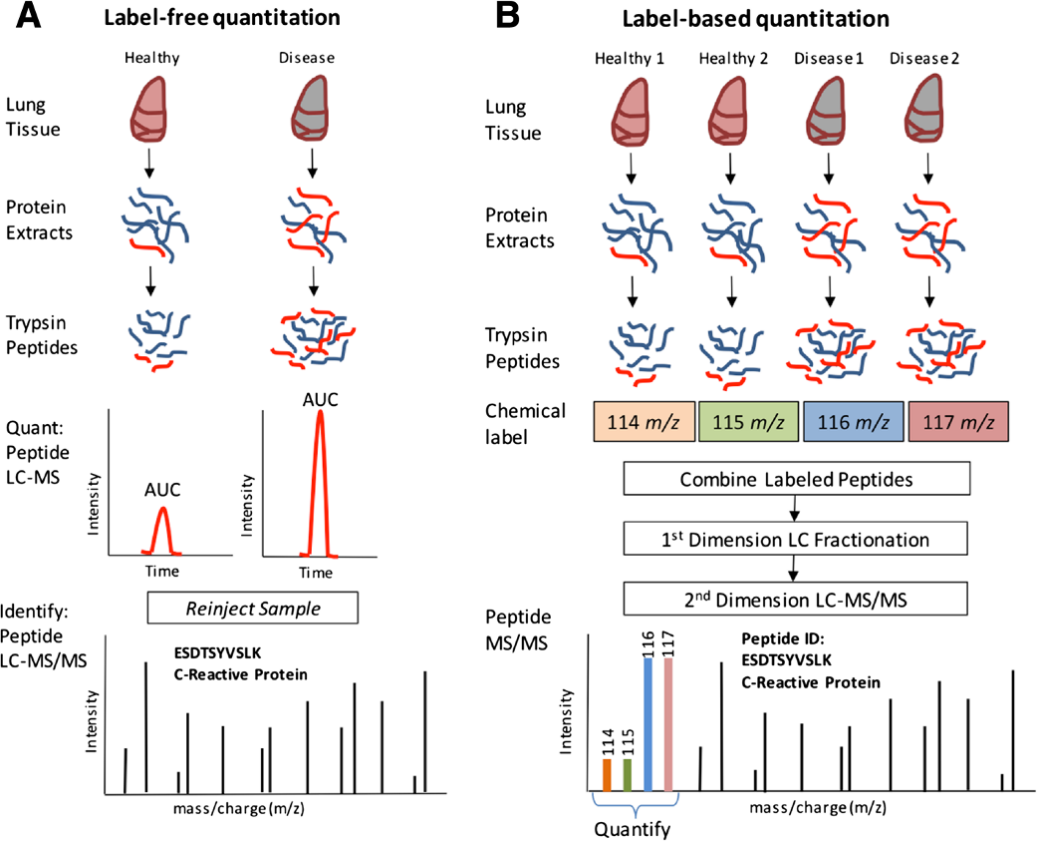
**Principles of quantitative proteomics. A)** Label-free quantitation performed by peptide peak area under the curve. Proteins are extracted from tissue, proteolytically digested into peptides and analyzed by liquid chromatography (LC)-MS. Analyte intensity versus retention time profiles are generated from which area under the curve (AUC) or summed peak intensities are calculated. Relative peptide amount in healthy versus disease sample is proportional to peak AUC or summed intensities. Targeted peptide identification is typically performed on a subsequent injection. **B)** Label-based quantitation with the iTRAQ® (isotope tagging for relative and absolute quantitation) 4plex workflow. Proteins from four individual samples are digested into peptides that are tagged with isobaric stable isotope labeled chemicals. Four chemical tags have 4 unique mass-to-charge (m/z) values that are produced during peptide tandem MS (MS/MS) and used for relative quantitation by relative peak intensity. Peptide fragment ions are used for peptide ID and protein inference.

Methods for protein quantitation in clinical samples can provide either relative or absolute quantitation. In the discovery phase of a project, relative protein quantitation is performed with the bottom-up, global approach from complex samples. Two discrete methods may be used: label-free [53] and differential isotopic labeling approaches [54],[55] (Figure 2). In both cases, equal amounts of protein extracts from multiple samples are processed by trypsin digestion and analyzed by LC-MS/MS.

Label free quantitation: Peptide counts per protein [56],[57] or peptide peak area under the curve generated during chromatographic separation [58],[59] define label free quantitation (Figure 2, panel A). Comparisons of peptide counts or peptide AUC across sample sets are performed with replicate measurements of each sample; higher peptide count or AUC represents higher relative abundance, when compared across samples. Label free quantitation is challenging due to the inherent variability in the spectral level data and extensive post processing required for minimizing this variability. This laborious approach has been used infrequently for studies in lung diseases.

Label based quantitation (SILAC, TMT, iTRAQ): The foundation of the peptide labeling approach is incorporation of heavy isotopes into peptides or proteins by metabolic or chemical labeling.

- *SILAC (stable isotope labeling by amino acids in cell culture*) technique incorporates stable heavy isotopes into proteins via labeled amino acids added as a growth supplement during cell culture [60]. Cells are grown in similar media without heavy isotope labeled amino acids under different conditions that establish the comparative assay. Proteins from `heavy’ and `light’ labeled and are digested into peptides, mixed and analyzed by mass spectrometry. Mass spectrometric peak intensities for the `heavy’ or `light’ peptides are used for relative protein quantitation among the select sample types. Equal amounts of protein are used for each sample under comparison so that any differences in relative peptide/protein amount measured by mass spectrometry reflect differences between samples, not starting protein amounts. Thus, sample preparation must be optimized to ensure accurate and consistent protein quantitation of the starting samples.
- TMT and iTRAQ: Differential labeling of protein extracts from discrete samples can be multiplexed with the commercial TMT (tandem mass tags) and iTRAQ (isobaric tagging for absolute and relative quantitation) amine-specific chemical reagent tags [61]-[63] (Figure 2, panel B). Comparison of protein expression levels of 2 – 10 sample types is made with heavy isotope-labeled functional groups of isobaric compounds that bind to peptide free amines [64]. These label-based methods allow for estimation of relative protein abundance [65].

#### Targeted proteomics

Mass spectrometry can be employed as a targeted assay for the detection and precise quantitation of limited number of biomolecules identified from discovery based experiments with selected reaction monitoring (SRM) MS [66],[67] or multiple reaction monitoring (MRM) MS assays. Protein detection or absolute protein quantification is achieved by selective measurement of peptides from proteolytic (e.g., tryptic) digestion of clinical samples on a specialized mass spectrometer, typically a triple quadrupole MS. The mass spectrometric acquisition method contains a list of the mass-to-charge values of the select peptides from the target protein(s) as well as the mass-to-charge values for one or more peptide fragment ions generated by tandem MS. The mass spectrometer acts as a selective mass-based detector for the chosen molecules; very low detection limits can be achieved, for instance, <10 fmol per molecule. MS measures peptides after separation by liquid chromatography. Chromatographic peptide peak integration is used for quantitation with the stable isotope dilution method using heavy isotope-labeled peptides as internal standards, which are spiked into the samples during work-up. The term multiple reaction monitoring (MRM) refers to an acquisition method for monitoring multiple peptide fragment ions per peptide as a measure of increasing specificity of detection for the select molecules. The sensitivity of SRM assays surpasses the sensitivity of data-dependent discovery based assays [68]. SRM methods provide a fast, cost effective way to validate biomarker candidates or quantitative proteins from large sample sets. Targeted analyses require significant method development but provide a means for absolute quantitation of proteins with a low coefficient of variance [69]-[71].

### Samples for lung proteomics

Proteomic studies begin with protein extraction from biological sample. Either tissue specimens and/or biological fluids can be used for proteomic investigations. Clinical-based samples, specifically body fluids, pose unique challenges for proteomics experiments due to the wide dynamic range of proteins typically present in most samples. Since MS is a concentration dependent technique, the molecules of highest concentration in a sample are detected preferentially over lower abundant species. The presence of `matrix’ biomolecules such as mucins (e.g, large MW glycoproteins) and surfactants (e.g., phospholipoproteins) in pulmonary fluids complicate sample preparation since they must be removed during initial sample preparation steps. Sample cleanup and preparation methods must be developed and validated for specific applications. The initial step of protein extraction from either the cells or body fluids is the most critical for achieving successful and reproducible outcomes, and is overall the most challenging step in a mass spectrometry-based proteomics experiment.

For lung diseases, including ARDS, it would be ideal to have lung tissue from an involved region for proteomic studies; however, lung biopsy specimens often are not available. Biological fluids that have been studied for extracellular proteins include plasma/serum. Using these biological fluids offers the benefit of repeated sampling but the lung specific signal likely is diluted. Consequently other body fluids such as sputum [72], epithelial lining fluid (ELF) [73] lung edema fluid [74], exhaled breath condensate [75] and bronchoalveolar lavage fluid (BALF) have been investigated.

#### Sputum

Sputum consists of expectorated secretions from the respiratory tract. In a study, Nicholas et al. studied sputum proteins from one healthy smoker using either 2-DE or SDS-PAGE followed by Gel LC MS/MS. By 2-DE over 600 features were present in the sputum, however only 61 proteins were identified when spots present in at least three replicate gels were excised and analyzed by MS/MS after *in-situ* trypsin digestion. Most of these proteins represented high abundance proteins previously reported in sputum, saliva, BAL and nasal lining fluid. In contrast, Gel LC-MS/MS provided extended coverage with identification of 191 human proteins, which also included low abundance proteins such as mucins, uteroglobin related protein etc. The authors reported striking similarity between the proteome of the sputum and BAL [76]. Gray et al. [72] investigated sputum from healthy controls and subjects with obstructive airways disease (asthma or COPD) and suppurative airway diseases (cystic fibrosis or bronchiectasis). These studies using top-down SELDI-TOF methodology identified approximately 50 (p-value <0.001) proteins peaks that differentiated healthy control subjects from patients with asthma or COPD and approximately 300 protein peaks (p-value <0.001) that differentiated healthy controls from subjects with bronchiectasis or CF. Calgranulin A, B and C were more abundant in bronchiectasis and CF and not seen in COPD or asthma. In this study, club cell secretory protein (CCSP) was present in lesser amount in both obstructive and suppurative lung diseases compared to healthy controls.

#### Bronchoalveolar lavage fluid

The epithelial lining fluid of the lung contains locally produced proteins that participate in a variety of different functions including defense mechanism, tissue remodeling, oxidant-antioxidant systems, inflammatory processes and cell growth. This fluid can be sampled directly by performing bronchoalveolar lavage. The proteins in BALF also may originate from diffusion from the serum; however comparison of serum and BALF proteomes demonstrates the presence of certain proteins at higher quantities in the BALF, suggesting alveolar and airway epithelial cells specifically secrete some of these proteins [77]. Thus, BALF is particularly attractive to investigate in pulmonary diseases such as ARDS as it reflects the fluid most proximate to the site of injury.

Two dimensional gel electrophoresis (2-DE) and LC-MS has been used for characterizing the protein expression in BALF [78]-[82]. One of the first studies mapping BALF proteins using 2-DE demonstrated mostly plasma proteins [82]. Subsequent studies using more sophisticated sample preparation technique have demonstrated a more comprehensive map of the BALF proteins [79],[80],[83] resulting in creation of a database of BALF proteins [81],[84]. The 2-DE map created by characterizing both individual and pooled BALF form subjects with different lung conditions has resulted in visualization over 1200 silver stained spots and identification of 900 proteins that include intact proteins or protein subunits and fragments [84]. However the major challenges in BALF proteomics are high salt and low protein content with wide dynamic range. Several of the sample preparation techniques used for 2-DE, such as desalting of the BALF, continue to be used for contemporary MS studies to address this issue. The removal of albumin [85] and other high abundance proteins that allows for investigating the lower abundance proteins, referred to as *deep proteome profiling*, has also improved identification of low abundance proteins [27][86],[87] and is a useful strategy for LC-MS based proteomics. Recent report by Goodlet et al. reviews studies applying shot-gun proteomics to BALF [88]. Our laboratory has optimized BALF sample preparation for semi-quantitative protein expression studies using iTRAQ® LC-MS/MS for patients with ARDS. Initial studies using removal of six high abundant proteins (albumin, transferrin, IgG, IgA, haptoglobin and antitrypsin) resulted in identification of only 93 proteins at a FDR of 5% (abstract presented at ASPEN lung meeting). Optimization of sample preparation that included careful selection of spin columns for desalting and concentration of the BALF, depletion of 14 high abundance plasma proteins - albumin IgG, α1-antitrypsin, IgA, IgM, transferrin, haptoglobin, α2-macroglobulin, fibrinogen, complement C3, α1-Acid glycoprotein (orosomucoid) , HDL (apolipoproteins A-I and A-II), LDL (mainly apolipoprotein B)- in combination of use of high resolution Orbitrap MS resulted in improved coverage with identification of 724 proteins at 1% global FDR [36]. With improvement in the tools available to researchers, it is likely that challenges with BALF such as high dynamic range, protein loss during sample preparation, and variable states of dilution during sampling will be overcome and a comprehensive database of BALF proteome will become available.

#### Serum or plasma

Plasma and serum is attractive due to ease of collection thus permitting serial measurements. This could be extremely valuable in ARDS to understand the pathological changes that occur during the development and recovery stages of this disease when lung specific biospecimens can be challenging to collect. Other advantages of identifying markers in serum or plasma include the ability to detect proteins with different tissue of origin such as the alveolar epithelial cells (SP-D, SP-A, RAGE), vascular endothelium (vWF), matrix metalloproteinase and mediators of inflammation [89]. However, barriers to successful plasma biomarkers include the high level of complexity of the proteome in addition to high abundance proteins limiting the systematic study of medium or low abundant proteins. Similar to BALF, immunodepletion of high abundance proteins has been used for plasma proteomics in ARDS [90],[91].

Other potential bio-fluids that could be investigated include urine, nasal lavage fluid, and pleural effusion fluid. However, currently there is limited evidence of the utility of these samples in the study of ARDS.

### Proteomics in ARDS

ARDS is acute respiratory failure with bilateral infiltrates due to permeability pulmonary edema resulting in hypoxia with a decrease in PaO_2_ to FiO_2_ ratio in absence of congestive heart failure [92]-[94]. ARDS continues to be associated with a relatively high mortality [95],[96]. American European Consensus Conference criterion used the term Acute lung injury (ALI) for milder form of ARDS [94] but Berlin definition has suggested to use mild ARDS instead of ALI [97]. Current knowledge is that ARDS is associated with an exuberant inflammatory response in the lung resulting in diffuse alveolar damage, surfactant dysfunction, epithelial and endothelial damage with loss of alveolar-capillary barrier and leakage of protein rich edema fluid into the alveolus that results in impaired gas exchange. Following the exudative phase the lung attempts to repair itself by proliferation of type II alveolar epithelial cells which then differentiated into type I alveolar epithelial cells and ultimately leading to regeneration of the alveolar epithelium and clearing of edema fluid and cellular debris form the alveolus. Proteomics studies have been used to provide novel insight to the mechanisms underpinning the development of and recovery from ARDS and also to discover biomarkers of the disease (Table 1).

**Table 1:** **Studies in ARDS using proteomics platforms**

Year	Proteomics methodology	Sample type	Number of subjects	Number of proteins identified	Reference
2004	2DE-MALDI/TOF	Plasma and Edema fluid in ARDS and Plasma and BALF in non-smoking healthy controls	ALI/ARDS = 16, Controls = 12	300 distinct protein spots and 158 proteins identified.	Bowler [74]
2006	SELDI-TOF and 2DE + MALDI TOF/TOF	BALF	ARDS = 11, Healthy nonsmoking controls = 33	Only differentially expressed proteins reported	De Torre [98]
2006	`Bottom-up’ proteomics with LC-MS/MS	BALF	ARDS = 3	226, 291 and 659 proteins for the three patients studied	Schnapp [35]
2008	2DE-MALDI TOF/TOF	BALF	ARDS day 1 = 7 ARDS Day 3 = 8 ARDS day7 = 5	991 protein spots seen. Only 80 protein spots analyzed by MS which represented 37 unique proteins	Chang Martin [38]
2013	MALDI TOF/TOF	Pooled plasma	Direct lung injury = 6, Indirect lung injury = 5, healthy controls = 15	132 proteins	Chen [90]
2014	iTRAQ Orbitrap LC-MS/MS	Pooled BALF	Early phase ARDS survivors = 7 Early phase ARDS non-survivors = 8 Late phase ARDS survivors = 7	724 proteins identified, 499 proteins quantified	Bhargava [36]

Initial attempts to study the proteome in ARDS were performed using gel-based platforms. First attempts at applying proteomics to ARDS were published by Bowler [74] where they studied plasma and edema fluid (EF) in 16 (age 55 ± 3) patients with ALI/ARDS (PF ratio 124 ± 15) and plasma and BALF in 12 normal non-smoking subjects (age 25 ± 5). Studies performed using 2-DE demonstrated 300 distinct protein spots in healthy volunteers. In healthy controls, the protein profile was globally similar except that there was some variability in the intensity of protein spots. Multiple isoforms of some proteins such as SP-A, IgA and IgM, were evident in the BALF. A few proteins were present only in the BALF and not in the plasma. Several proteins such as albumin, haptoglobulin, IgG, fibrinogen, apolioporotien, clusterin-sulfated glycoprotein-2, transferrin, retinol binding protein, and transthyretin all had more intense staining in the plasma than BALF. In patients with ALI/ARDS the protein spot profile could be grouped into three patterns when compared to controls- 1) increased protein intensity, 2) decreased protein intensity or 3) modified expressions due to presence of post-translational modifications. The spots with increased relative intensity in EF of all ALI subject were of albumin, transferrin, IgG and clusterin. In contrast, SP-A was seen in the BALF for all normal subjects but only one patient with ALI/ARDS. Similarly, alpha-1-anti trypsin was identified in all normal subjects but only half of ALI/ARDS patient’s. Haptglobin and orosomucoid appeared to be have undergone post-translational modification in ALI/ARDS. The authors concluded that proteomics has potential to study the air space in patient’s with ALI/ARDS with the ability to identify post-translational modifications that would not be possible with other techniques.

In another study de Torre et al. [98] used top-down SELDI-TOF methodology and 2-DE with MALDI-TOF MS to identify BALF protein profile differences in ARDS compared to normal subjects. Study subjects included 11 cases within 72 hours of meeting the ARDS criterion and 33 healthy nonsmoking subjects challenged by either saline or endotoxin for induction of local lung inflammation followed by BAL in 6, 24 and 48 hours. Their studies revealed the presence of differentially expressed proteins in endotoxin challenged compared with saline challenged subjects. Three peaks at 14,18 and 28 kDa were more prominent in the endotoxin challenged subjects. The inflammation persisted at 24 hours but decreased at 48 hours after the endotoxin challenge. The pattern from ARDS cases were similar to that seen at 6 hours after the endotoxin challenge with increase in the 14 and 28 kDa peak intensity. Subsequent 2-DE combined with in-gel trypsin digestion with MALDI-TOF MS identified increased level of apolipoprotein A1, S100-A8 and A9 in subjects challenged with endotoxin and ARDS.

Other studies have used MS for characterizing global changes in BALF in patients with ARDS. In a study Chang et al. [38] performed DIGE followed by MS-based proteomics in combination with *in silico analysis* to characterize serial changes in ARDS BALF at day 1 (n = 7), day 3 (n = 8), and day 7 (n = 5) and compared these to normal volunteers (n = 9). Protein separation using DIGE showed an average of 991 protein spots in each group of patients. Of these 991 protein spots, 80 spots of interest were chosen for further study using tandem MALDI-TOF/TOF resulting in identification of 37 unique proteins that represented opsonins, antioxidants, basement membrane proteins, coagulation proteins and acute phase reactants. Twenty-two of these proteins were differentially expressed over time compared to controls. This type of study lends itself to functional analysis and Gene Ontology of these 22 proteins demonstrated processes involved in inflammation, response to microbials and response to stress/injury. An advantage of this approach is a sophisticated network analysis that revealed complex and redundant dynamic changes suggesting the complex nature of protein changes in ARDS. Several of the proteins that were previously known to be critical in ARDS such as TNF alpha, IL-1beta, LBP, p38MAPK were central hubs in the identified networks in this study. Time course network analysis showed temporal dynamic changes. Compared to controls, on day one of the ARDS diagnosis there were increases in complement proteins, annexin A3, S100 protein, antiproteases, actin and extracellular matrix proteins in the BALF. In contrast, surfactant protein-A, annexin A1, fibrinogen and fatty acid binding protein were decreased in ARDS compared to control. Differences between day one and day three of ARDS were less dramatic though complement C3 and preredoxin-2 showed a major difference. By day seven, there was evidence of regeneration of the lung epithelium, decreased cellular injury, cell turnover and resolution of lung injury.

Our laboratory has used label based quantitative `bottom-up’ proteomics (iTRAQ® Orbitrap LC-MS/MS) and characterized protein expression form ARDS patients who had BALF collected either in early phase of ARDS (day 1-7 after intubation) or late phase (≥8 days post intubation) [36]. The goal of these studies was to identify differentially expressed proteins in early phase survivors when compared to early phase non-survivors and determine the biological processes that are lacking or over-expressed in the two groups with divergent outcomes. We identified 724 proteins (FDR≤1) of which 499 proteins had quantitative data available. The proteins that were overexpressed in early phase survivors represent six ontologies- three related to coagulation, fibrinolysis and wound healing, two related to iron and cation homeostasis and one related to immune system activation. In contrast, the early phase non-survivors had a signature of collagen deposition, carbohydrate catabolism and actin cytoskeleton organization. Proteins that are differentially expressed in these biological processes could be potential biomarkers for prediction of outcomes in ARDS. In this study when early phase survivors were compared to late phase survivors, biological processes that were activated in late phase were cell migration and actin filament based processes suggesting dynamic changes in the BALF occur in ARDS subjects who survive. The processes that get activated in late phase ARDS survivors could be potential targets to design novel therapeutics and be manipulated in early ARDS in patients predicted to have poor outcomes.

In a recent study, pooled plasma from patients with ARDS due to direct lung injury (n = 6), indirect lung injury (n = 5) and normal controls (n = 15) were analyzed using semi-quantitative proteomics by iTRAQ with MALDI-TOF tandem MS [90]. Despite depletion of albumin and IgG, the proteome coverage in this study was limited with identification of 2429 peptides with only 132 non-redundant inferred proteins. Of these 132 proteins only eleven proteins were differentially expressed in ARDS compared to controls, seven up regulated and four down regulated. The canonical pathways represented by these proteins were liver X receptor/retinoid X receptor (LXR/RXR) and farnesoid X receptor (FXR)/RXR activation, clathrin-mediated endocytosis signaling, atherosclerosis signaling, IL-12 signaling and production in macrophages, nitric oxide and reactive oxygen species production in macrophages, and complement system signaling. Due to the limited protein coverage and relatively small number of differentially expressed proteins, any protein pathway inference requires further investigation. This study highlights the ongoing challenges of plasma/serum proteomics due to wide dynamic rage and lack of deep proteome coverage in these biofluids.

In addition to BALF and plasma, exhaled breath condensate has been studied by SDS gel separation in combination with MALDI-TOF in patients with respiratory failure [99]. A high level of cytokeratin 2 and 10 was associated with increased peak inspiratory pressure; PEEP and ARDS score suggesting that cytokeratins correlated with mechanical stress. These studies are examples how extended proteome coverage of lung biospecimens by different proteomics platforms and computational tools can lend new insights into the pathobiology of ARDS.

## Conclusion and future of proteomics in lung diseases

Significant strides have been made in several techniques that are available for large-scale studies of proteins in biological systems. Mass spectrometer based proteomics has evolved from the ability to identify proteins present in a complex mixture to its current state where both label free and label-based methodologies can provide quantitative information regarding proteins with high precision. Label based methodologies are currently used more widely but one of the limitation of these techniques is co-isolation of more than one peptide for tandem MS which would provide imprecise quantification. Label free quantification with SRM and MRM requires prior information of the peptide behavior of the proteins of interest. Targeted proteomics with SRM or MRM is also dependent on sample processing prior to LC-MS and thus precludes measurement of low abundance proteins. Some of the newer techniques that implement unbiased data independent acquisition by mass spectrometry followed by targeted data extraction such as SWATH-MS (**S** equential **W** indowed data independent **A** cquisitionof the **T** otal **H** igh-resolution **M** ass **S** pectra) [100] offer promise for high throughput precise quantification of large number of proteins. Sophisticated bioinformatics algorithms are also being developed (inSeq) [101] which implement real time assignment of the spectral matches allowing for improved accuracy of quantitation and also improved localization of post translational modifications. Better understanding of post-translational modifications will allow more comprehensive mapping of networks and pathways implicated in certain diseases. In addition to advanced algorithms for protein inference, there is a major opportunity to understand the systems that are contributing to a disease state by integrating proteomics with other platforms such as next generation sequencing and small molecule studies using metabolomics. This `multi-dimensional data integration’ would be key to develop targeted therapies for complex conditions like ARDS.

## Authors’ contributions

All authors have read and approve the final manuscript. MB: Review of literature, manuscript draft and revision. LAH: Review of literature, manuscript draft and revision. CHW: Manuscript draft and revision. DHI: Manuscript draft and revision.

## Authors’ original submitted files for images

Below are the links to the authors’ original submitted files for images. Authors’ original file for figure 1 Authors’ original file for figure 2

## Abbreviations

ARDS: Acute respiratory distress syndrome
MS: Mass spectrometer
1D: 1-dimensional
2D: 2-dimensional
SDS-PAGE: Sodium dodecyl sulfate polyacrylamide gel electrophoresis
MALDI: Matrix-assisted laser desorption ionization
ESI: Electrospray Ionization
SPE: Solid phase extraction
MALDI-TOF: Matrix-assisted laser desorption ionization-time of flight
SELDI-TOF: Surface-enhanced laser desorption ionization time of flight
LC: Liquid chromatography
LC MS/MS: LC tandem MS
AUC: Area under the curve
SIALC: Stable isotope labeling by amino acids in cell culture
TMT: Tandem mass tags
iTRAQ: Isobaric tagging for absolute and relative quantitation
SRM: Selected reaction monitoring
MRM: Multiple reaction monitoring
BALF: Bronchoalveolar lavage fluid
2-DE: Two dimensional gel electrophoresis
IEF: Isoelectric focusing
Ig: Immunoglobulin (IgG ,IgA, IgM)
SP: Surfactant protein (SP-A, SP-B, SP-D)
LDL: Low density lipoproteins
HDL: High density lipoproteins
RAGE: Receptor for advanced glycation end products
vWF: von-willibrand factor
FiO_2_: Fraction of inspired oxygen
ALI: Acute Lung Injury
IGFBP-3: Insulin-like growth factor binding protein-3
PEEP: Positive end expiratory pressure
LXR: Liver X receptor
RXR: Retinoid X receptor
FXR: Farnesoid X receptor
IL: Interleukin
FDR: False discovery rate
SWATH-MS: Sequential windowed data independent acquisitionof the total high-resolution mass spectra
