# Supplementary figures and images for: Application of clinical proteomics in acute respiratory distress syndrome
Source: Clin Transl Med. 2014 Oct 15;3:34. doi: 10.1186/s40169-014-0034-1 (PMC4883989; doi:10.1186/s40169-014-0034-1)

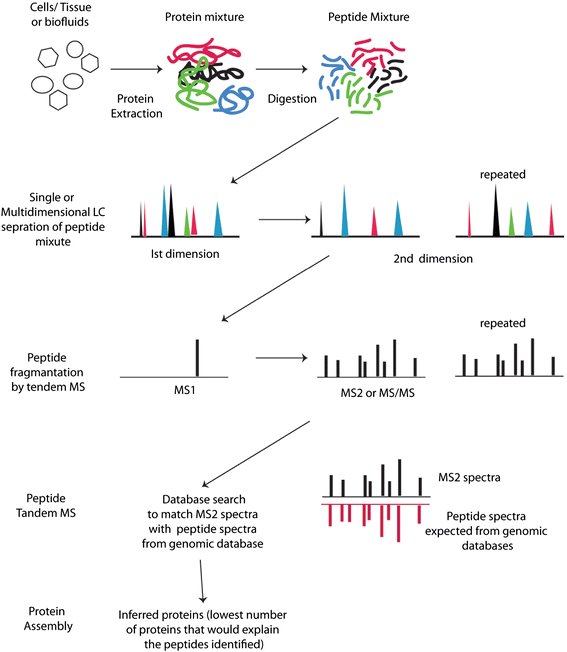

Supplement: Supplementary file 1 — Authors’ original file for figure 1 [file 40169_2014_34_MOESM1_ESM.gif]

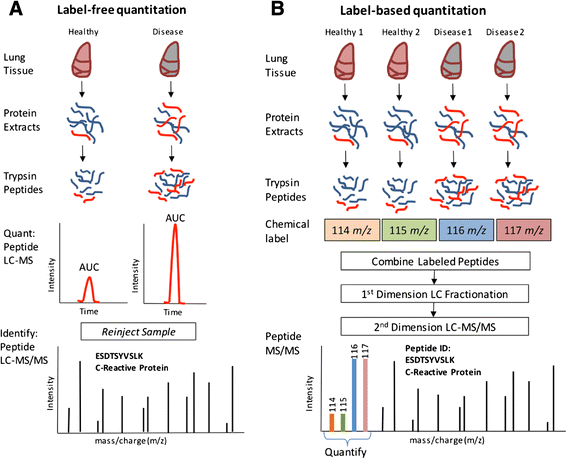

Supplement: Supplementary file 2 — Authors’ original file for figure 2 [file 40169_2014_34_MOESM2_ESM.gif]
